# Supplementary material for: Persistent Mesodermal Differentiation Capability of Bone Marrow MSCs Isolated from Aging Patients with Low-Energy Traumatic Hip Fracture and Osteoporosis: A Clinical Evidence
Source: Int J Mol Sci. 2024 May 12;25(10):5273. doi: 10.3390/ijms25105273 (PMC11120803; doi:10.3390/ijms25105273)
Supplement: Supplementary file 1 [file ijms-25-05273-s001.zip › ijms-2915377-supplementary.pdf]

Supplementary information

Table S1. Clinical information and bone marrow samples of patients enrolled in the current study.

	**Case 1**	**Case 2**	**Case 3**	**Case 4**	**Case 5**	**Case 6**	**Case 7**
**Sample code**	OP-001	OP-002	OP-003	OP-004	OP-005	OP-006	OP-007
**Extraction date**	2019.5.16	2019.5.31	2019.7.3	2019.7.25	2019.7.30	2019.9.19	2019.10.8
**Pathogen**	Pass	pass	pass	pass	HBVs:+	-	Anti-HCV:+
**BM vol.**	40ml	27ml	40ml	25ml	35ml	-	27.5ml
**Extraction site**	L. Hip	R. Hip	R. Hip	L. Hip	R. Hip	L. Hip (terminate)	L. Hip
**Age**	95	79	76	83	73	67	85
**Gender**	M	F	F	F	F	F	M
**Fracture times/site**	1 (L)FNF	2 (L)FNF ->(R)ITF	2	1 (L)hip ITF	1	-	1

	**Case 8**	**Case 9**	**Case 10**	**Case 11**	**Case 12**	**Case 13**	**Case 14**
**Sample code**	OP-008	OP-009	OP-010	OP-011	OP-012	OP-013	OP-014
**Extraction date**	2019.10.24	2019.11.8	2019.12.13	2020.2.13	2020.3.30	2020.4.14	2020.4.29
**Pathogen**	Anti-HCV:+	pass	pass	Anti-HCV:+	pass	pass	pass
**BM vol.**	27.5ml	~25ml	23ml	~45ml	~25ml	~37.5ml	~35ml
**Extraction site**	R. Hip	L. Hip	L. Hip	L. Hip	L. Hip	L. Hip	R. Hip
**Age**	85	56	77	97	90	90	72
**Gender**	F	F	M	F	M	F	M
**Fracture times / site**	1	1 (L)FNF	2 (R)ITF -> (L)ITF	1	1	1	1

	**Case 15**	**Case 16**	**Case 17**	**Case 18**	**Case 19**
**Sample code**	OP-015	OP-016	OP-017	OP-018	OP-019
**Extraction date**	2020.5.13	2020.5.19	2020.6.12	2020.6.30	2020.9.15
**Pathogen**	HBVs:+	Pass	pass	pass	pass
**BM vol.**	~40ml.	~42ml	~40ml	35ml	~50ml
**Extraction site**	L. Hip	L. Hip	L. Hip	R. Hip	R. Hip
**Age**	57	75	89	85	92
**Gender**	F	F	F	M	F
**Fracture times/site**	1	1	1	1	1

Table S2. Details of BMSCs collected from patients enrolled in the current study and patients' disease background

Sample	Age (gender)	BMI	Disease history	Pre-surgery Hb	Diagnosis	Surgery	BM collection date	BMD date	BMD (L-spine)	BMD (FN)
OP-001	95 (M)	19.72	L1,L3 compression fx<br>HTN, arrhythemia	13.8	Lt FNF	bipolar	2019.05.16	2019.07.12	+1.9	Rt FN -1.8
OP-002	78 (F)	23.56	Lt FN s/p bipolar (2018.11.29)<br>HTN, s/p thyroidectomy<br>Osteoporosis under denosumab	11.2	Rt ITF	ORIF	2019.05.31	2018.12.26	-3.1	Rt FN -3.1
OP-003	76 (F)	23.11	HTN<br>L-spine s/p	12.1	Rt FNF	bipolar	2019.07.03	-		
OP-004	83 (F)	23.50	DM, HTN, thalassemia<br>Osteoporosis (bonviva)	8.9	Lt ITF	ORIF	2019.07.25	2014.10.20<br>2020.07.29	-3.7<br>-4.7	Rt FN -3.1<br>Rt FN -4.4
OP-009	56 (F)	19.47	Depression, nursing home<br>anemia	9.6	Lt FNF	bipolar	2019.11.06	2019.12.06	-2.5	Rt FN -2.9
OP-010	77 (M)	22.31	Osteoporosis under Bonviva<br>Rt ITF s/p (2018.08.17)<br>Smoking(+)	12.6	Lt ITF	ORIF	2019.12.13	2018.10.24	-0.0	Lt Thip -2.7
OP-014	72 (M)	23.60	HTN, DM<br>L1 wedge deformity<br>Smoking (+)	14.8	Rt ITF	ORIF	2020.04.28	-		
OP-016	75 (F)	20.55	T11 compression fx	9.6	Lt FNF	bipolar	2020.05.18	-		
OP-017	89 (F)	19.65	dementia	13.2	Lt FNF	bipolar	2020.06.12	2020.06.15	-1.8	Rt FN -4.3
OP-018	85 (M)	22.52	T12/L1 compression fx<br>HTN<br>Smoking (-)	12.9	Rt FNF	bipolar	2020.06.29	2020.07.03	0.6	Lt FN -2.4
OP-019	92 (F)	20.44	DM, HTN, Alzheimer dz	10.0	Rt ITF	ORIF	2020.09.14	2020.09.17	-2.4	Lt FN -3.8
THCTB006	21 (M)		Smoking		Rt FSF		2017.01.11			
THCTB008	22 (M)		HCV(+), smoking		Rt FSF		2017.04.08			
T2B-007	24 (M)				Rt FSF		2018.12.20			

Abbreviation: not determined (nd), fixation (fx), hypertension (HTN).

Table S3. Details of BMSCs collected from the young, healthy donors

	**Case 1**	**Case 2**	**Case 3**
**Sample code**	THCTB-006	THCTB-008	T2B-007
**Extraction date**	2017.01.11	2017.04.08	2018.12.20
**Pathogen**	Pass	pass	pass
**BM vol.**	40 ml	40 ml	40 ml
**Extraction site**	R. femur	R. femur	R. femur
**Age**	21	22	24
**Gender**	M	M	M
**Fracture times / site**	1<br>(R)femur fracture, car accident	1<br>(R)femur fracture, car accident	1<br>(R)femur fracture, car accident
**Note**	Smoking	Smoking	-

Table S4. Details of selected primary antibodies and isotypes for FACS analysis.

Antibody name	Clone	Description	Cat no	Lot no	Brand
HLA-DR-FITC	G46-6	Mouse IgG2a	555811	1103331	BD Pharmingen
CD14-FITC	M5E2	Mouse IgG2a	555397	0357884	BD Pharmingen
Isotype-FITC	IgG2a	Mouse IgG2ak	555573	1144955	BD Pharmingen
HLA-ABC-FITC	G46-2.6	Mouse IgG1	555552	9105740	BD Pharmingen
CD45-FITC	HI30	Mouse IgG1	555482	1285678	BD Pharmingen
CD19-FITC	HIB19	Mouse IgG1	555412	9308489	BD Pharmingen
CD105-FITC	SN6	Mouse IgG1	MCA1557F	158807	Bio-Rad
Isotype-FITC	MOPC-21	Mouse IgG1	555748	1054855	BD Pharmingen
CD34-PE	581	Mouse IgG1	555822	0128396	BD Pharmingen
CD90-PE conjugated	5E10	Mouse IgG1,kappa	555596	2026304	BD Pharmingen
CD73-PE	AD2	Mouse IgG1,kappa	550257	1075157	BD Pharmingen
Isotype -PE	MOPC-21	Mouse IgG1	555749	8242609	BD Pharmingen

A

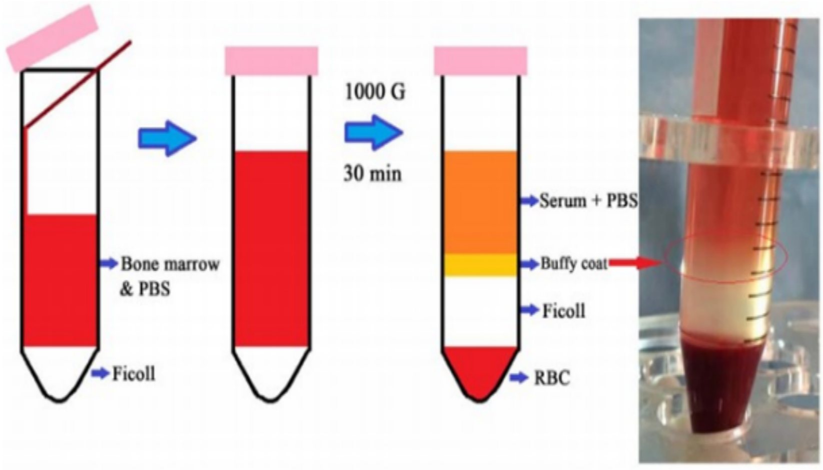

B

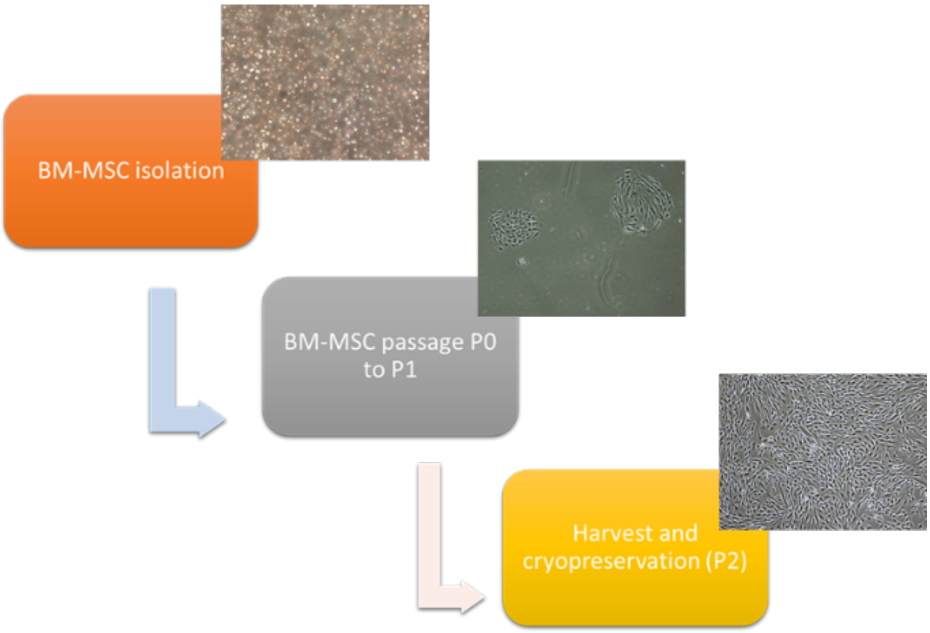

Figure S1. Isolation of bone marrow stem cells by Ficoll gradient centrifugation. **A** Ficoll gradient centrifugation was conducted in 4 °C. **B** Cells in the buffy coat layer were further collected, counted, and plated in the culture dish using α-MEM containing 10% FBS and the P2 BMSCs were cryo-preserved for subsequent experiments.

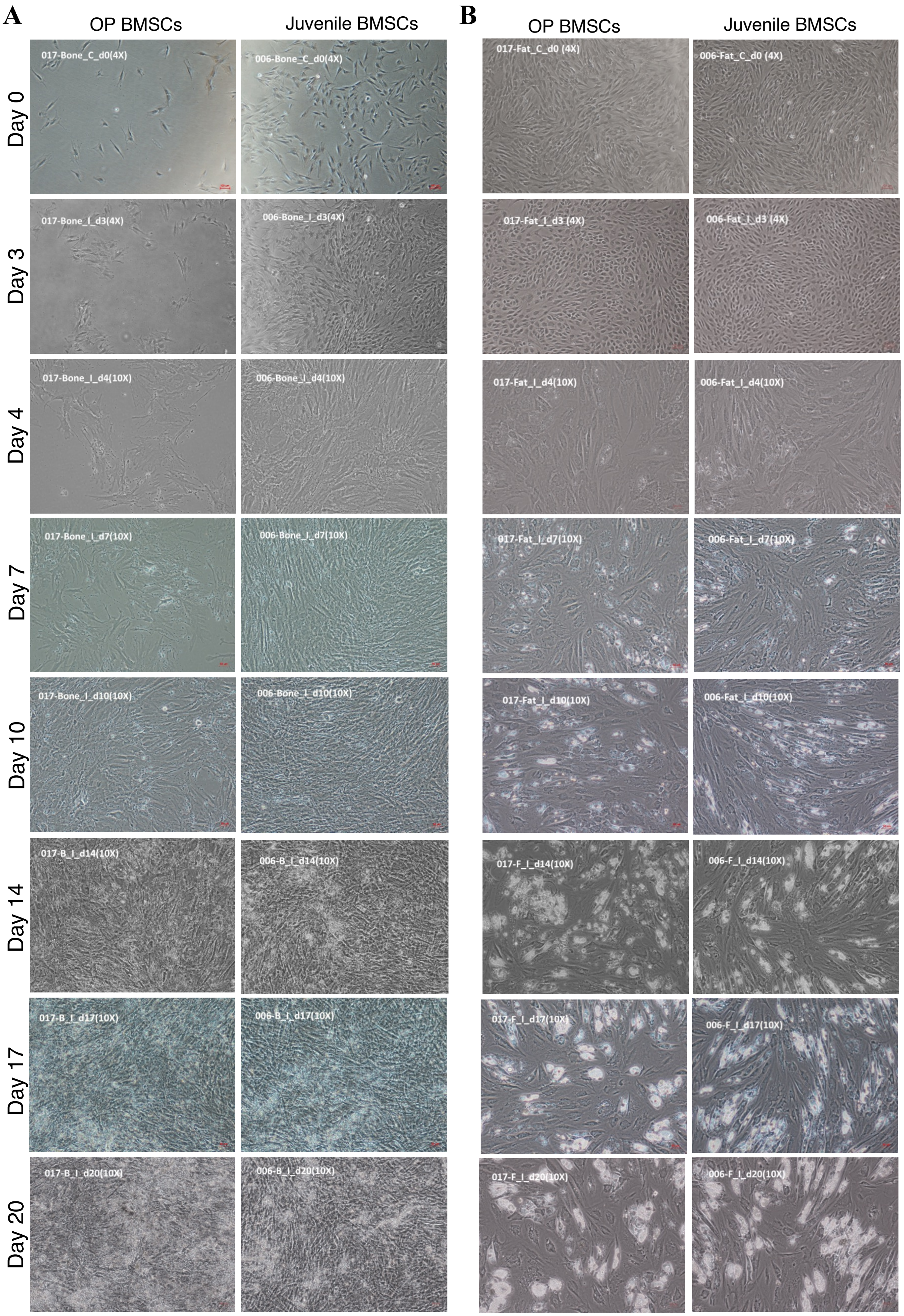

Figure S2. Representative images examined the longitudinal cultivation of the osteogenic and adipogenic differentiation capability of BMSCs retrieved from OP patients and healthy donors. **A** osteogenic differentiation. **B** adipogenic differentiation. Bright-field images. Magnification: 100×.
